# Difficult to control atopic dermatitis

**DOI:** 10.1186/1939-4551-6-6

**Published:** 2013-03-14

**Authors:** Ulf Darsow, Andreas Wollenberg, Dagmar Simon, Alain Taïeb, Thomas Werfel, Arnold Oranje, Carlo Gelmetti, Ake Svensson, Mette Deleuran, Anne-Marie Calza, Francesca Giusti, Jann Lübbe, Stefania Seidenari, Johannes Ring

**Affiliations:** 1 Department of Dermatology and Allergy Biederstein, Technische Universität München, Munich, Germany; 2 ZAUM – Center for Allergy and Environment, Munich, Germany; 3 Department of Dermatology and Allergy, Ludwig-Maximilians-University Munich, Munich, Germany; 4 Department of Dermatology, Inselspital, Bern University Hospital, University of Bern, Bern, Switzerland; 5 Service de Dermatologie, Hopital St André, Bordeaux, France; 6 Hautklinik Linden, Deptartment of Dermatology MHH, Hannover, Germany; 7 Department of Pediatrics (Pediatric Dermatology Unit), ERASMUS MC, Rotterdam, The Netherlands; 8 Department of Pathophysiology and Transplantation, University of Milan, Ospedale Maggiore Policlinico, Milan, Italy; 9 Department of Dermatology, University Hospital UMAS, Malmö, Sweden; 10 Department of Dermatology, Aarhus University Hospital, Aarhus, Denmark; 11 Clinique de Dermatologie, Hôpital Cantonal Universitaire, Genève, Suisse, Switzerland; 12 Department of Dermatology, University of Modena and Reggio Emilia, Modena, Italy

**Keywords:** Atopic dermatitis, Eczema, Therapy, Guideline

## Abstract

Difficult to control atopic dermatitis (AD) presents a therapeutic challenge and often requires combinations of topical and systemic treatment. Anti-inflammatory treatment of severe AD most commonly includes topical glucocorticosteroids and topical calcineurin antagonists used for exacerbation management and more recently for proactive therapy in selected cases. Topical corticosteroids remain the mainstay of therapy, the topical calcineurin inhibitors tacrolimus and pimecrolimus are preferred in certain locations. Systemic anti-inflammatory treatment is an option for severe refractory cases. Microbial colonization and superinfection contribute to disease exacerbation and thus justify additional antimicrobial / antiseptic treatment. Systemic antihistamines (H1) may relieve pruritus but do not have sufficient effect on eczema. Adjuvant therapy includes UV irradiation preferably of UVA1 wavelength. “Eczema school” educational programs have been proven to be helpful.

## Introduction

Atopic dermatitis (AD, atopic eczema, eczema) is an inflammatory, chronically relapsing, and intensely pruritic skin disease occurring often in families with atopic diseases (atopic dermatitis, bronchial asthma and/or allergic rhino-conjunctivitis) [1]. Less than 10% are regarded as severe cases because of disease intensity and extent (SCORAD > 40) or refractory to treatment. Reasons for severe courses of AD are based on individual (e.g. genetic, barrier function, allergies) risc factors and sometimes on therapeutic problems like misunderstandings with regard to topical treatment. Management of exacerbated AD is a therapeutic challenge, as it requires efficient short-term control of acute symptoms, without compromising the overall management plan that is aimed at long-term stabilization, flare prevention, and avoidance of side effects.

**Figure 1:**
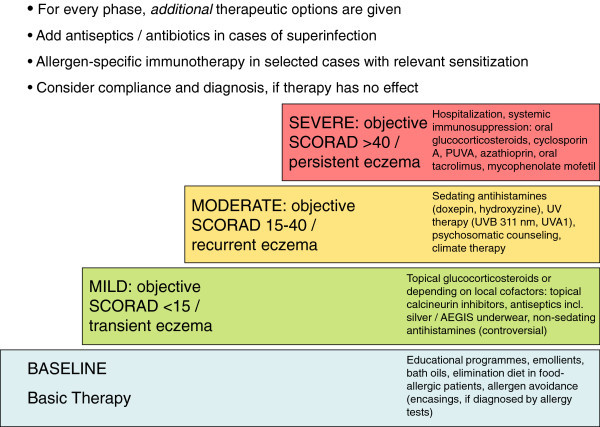
Recommendation for treatment of adult eczema / atopic dermatitis according to graded severity (modified from 44).

Exacerbations may sometimes uncover relevant provocation factors, for example contact allergy, or infection. This synopsis on refractory AD excludes the general principles of AD treatment like basic skin care and management of food allergy, as these main strategies are published elsewhere in guidelines [2]. Nevertheless, these basic rules have to be included in the management of all severe cases, too. Figure 1 summarizes the general treatment options.

### Contact allergy

The role of contact allergy in AD patients is frequently underestimated [3,4]. The frequency of contact sensitization in AD, ranging from 41% to 64% according to recent observations, supports the importance of systematic patch testing in atopic patients, adults and children. The most common contact sensitizers are metals, fragrance, preservatives, dyes, neomycin, and lanolin, but contact allergy to topical glucocorticosteroids is also not rare in AD patients.

Contact sensitization may worsen the skin condition of AD patients and influence the course of the disease. Moreover, sensitized AD subjects may respond to very low concentrations of contact allergens, because of their impaired skin barrier function and hyper-reactivity to irritant stimuli enhancing contact reactions.

### Topical anti-inflammatory treatment

#### Topical treatment

The use of wet-wrap dressings with (diluted) corticosteroids for up to 14 days (usually applied for 3 days) is a safe crisis intervention treatment of severe and/or refractory AD with temporary systemic bioactivity of the corticosteroids as the only reported serious side-effects [5,6]. By tradition, anti-inflammatory topical therapy has been administered to lesional skin only and has been stopped or tapered down once visible lesions were cleared. This traditional, reactive approach has in the last years been challenged by the proactive treatment concept, which is defined as a combination of pre-defined, long-term, low dose, anti-inflammatory treatment applied to previously affected areas of skin in combination with liberal use of emollients on the entire body and a predefined appointment schedule for clinical control examinations [7,8].

#### Corticosteroids

Topical corticosteroids (TCS) are a first line anti-inflammatory treatment, applied on inflammatory skin lesions according to needs (pruritus, sleeplessness, new flare). Proactive therapy for flare prevention is also possible with TCS.Topical corticosteroids are grouped by potency, which should be known to prescribers. Potent and very potent corticosteroids (group III and IV) are more likely to cause depression of adrenal function than group I (mild) and II (moderate strength) treatments, but their systemic effects will decrease more quickly due to more rapid restitution of the skin barrier [9,10]. However, potency ranking of topical steroids in some countries including the US is the reverse of the European system with class I (superpotent) to class VII (low potency). For severe AD, group II and group III (EU system) glucocorticosteroids are recommended. Itch is the key symptom for evaluation of response to treatment, and tapering should not be initiated before the itch has disappeared. Dose tapering should be gradual in order to avoid withdrawal rebound. Tapering strategies consist in using a less potent corticosteroid on a daily base, or keeping a more potent one while reducing the frequency of application (intermittent regimen).

#### Topical calcineurin inhibitors

Both topical calcineurin inhibitors (TCI), tacrolimus ointment (0.1% and 0.03%) and pimecrolimus cream (1%), are licensed for topical eczema treatment. Various aspects of these drugs have been reviewed in detail [11]. The efficacy of both formulations has been demonstrated against placebo in clinical trials for short term [12,13] and long term use of these substances [14,15]. In addition, proactive tacrolimus ointment therapy has been shown to be safe and effective for up to one year in reducing the number of flares and improving the quality of life in adult patients and children [16,17]. The anti-inflammatory potency of 0.1% tacrolimus ointment is similar to a corticosteroid with intermediate activity [18], while the latter is clearly more active than 1.0% pimecrolimus cream [19].

#### Antihistamines

Systemic antihistamines (anti H1) are widely used in acute flares against itch, however few controlled studies are available [20]. Antihistamines may be helpful in decreasing pruritus and permit sleep during flares. In this setting, sedative anti H1 molecules such as hydroxyzine are recommended by clinicians as helpful. Concerning the newer non-sedating H1R specific antihistamines, controlled studies did not show significant effects on eczema.

#### Anti-bacterial and antimycotic therapy

A number of defects in innate and adaptive immunity may explain the high rate of cutaneous colonization with *Staphylococcus aureus* (up to 90% in moderate to severe eczema) in AD [21]. There is evidence that a decrease in microbiome diversity is associated with an increased colonization with *S. aureus* as well as increase in disease activity. Antibiotic eradication of *S. aureus* may therefore not always be an appropriate long-term strategy, especially with regard to the increasing prevalence of antibiotic resistance [22-24]. In particular topical antibiotics should not be used for longer periods in the treatment of AD. There is evidence for an association of *S. aureus*-derived superantigens with disease exacerbation [25,26], supporting early observations that the density of *S. aureus* colonization in AD is significantly correlated with clinical severity [27], and that patients with severe AD may improve (but not be cured) by anti-staphylococcal treatment [28]. In general, improvement of eczema by anti-inflammatory therapy (i.e. TCS, TCI, UV) decreases *S. aureus* colonization.

Other secondary infections, such as yeasts, dermatophytes, and streptococcal infections have also been implicated as disease factors in AD (for a review, see [22]). Intense, fleshy erythema in skin folds of children with a flare of AD may warrant a search for streptococcal skin infection. In general, signs of secondary infections should be treated if present. Ketoconazole and ciclopiroxolamine are proposed for topical treatment of “head and neck” AD, often associated with *Malassezia sympodialis* superinfection [29,30].

#### Phototherapy

Except UVA1, which was shown to be effective in managing AD flares, phototherapy is not indicated in the acute stages of AD, but apt to treat chronic, pruritic, lichenified forms. However, it should not be prescribed in patients who experienced a worsening of their dermatosis during sun exposure. Usually, phototherapy is part of a total treatment plan in addition to topical anti-inflammatory and antimicrobial therapy. As second-level treatment it is used especially in adults. Phototherapy in children younger than 12 years should not be applied under normal circumstances.

Present UV sources include equipments able to emit selective spectra of radiations

– Broadband UV (UVA+UVB = 290–400 nm)

– Narrow-band UVB (nbUVB = peak:311–313 nm)

– UVA1 (340–400 nm).

nbUVB has been indicated for chronic-moderate forms of AD [31] and is currently preferred to broadband UV because it is less erythemogenic, while high dose UVA1 has been prescribed for more severe phases [31].

### Systemic anti-inflammatory therapy

Non-response to adequately applied topical therapy is rare, and systemic anti-inflammatory treatment should be limited to severe cases in which the potential of topical treatment (or of patient compliance) has been exhausted. An actual overview of the different options has been published [32]. Corticosteroids are rapidly effective, but should only be used for a few weeks, for severe acute exacerbations, because of the many long term side-effects. In severe chronic cases consider starting another systemic anti-inflammatory therapy while tapering the corticosteroid.

The usefulness of both cyclosporin (3–5 mg/kg/day) and azathioprine (2.5 mg/kg/day) has been well documented in clinical trials with children and adults [33-36]. Cyclosporin A therapy is rapidly effective, but has a narrow therapeutic index and requires a close follow-up of renal function. It is an approved substance for systemic treatment of AD in many countries and is frequently used for systemic immunosuppressive therapy in AD.

Azathioprine has a slower onset of action and is not always well tolerated. Low TPMT (thiopurinemethyltransferase) activity is associated with an increased myelotoxicity of azathioprine, but patients at risk can be identified by pre-treatment screening for TMTP activity [35].

Mycophenolate mofetil (2g/day) seems to offer a comparatively more favourable safety profile and its usefulness in severe AD is documented in both prospective and retrospective studies [37-39], but remains to be assessed in larger randomized trials.

Methotrexate is used by many clinicians as an alternative treatment. Only a few studies have documented its effect and more randomised trials are needed [40].

Biologic agents (biologicals) present new therapeutic tools in the treatment of recalcitrant AD. They specifically target inflammatory cells and mediators, respectively, and thus may inhibit pathogenically relevant pathways. A number of case reports and pilot studies have been published recently, however representative, randomized, placebo controlled studies evaluating the efficacy and safety of biologicals in AD are still not available. Approaches resulting in reduced T cell activation using agents such as alefacept (fusion protein of lymphocyte function antigen (LFA)-3 (CD58) and immunoglobulin (Ig)G, rituximab (anti-CD20 antibody) and efalizumab (anti-CD11a antibody, no longer available) have been shown to be effective in selected patients with moderate to severe AD and were mentioned in guidelines [2,41-44].

### Educational programs and counselling

In the last decade, education programs for patients and parents were established in different countries in Europe, but also in North and South America (see http://www.opened-dermatology.com). Standardized interdisciplinary programs involving dermatologists, paediatricians, psychologists / psychosomatic counsellors, and dietary counselling have been demonstrated to support the improvement of subjective and objective symptoms, and optimize medication use in patients, and result in a significant gain in quality of life [45]. Participation in one of these programs is highly encouraged.

## Competing interests

U. Darsow has been speaker, investigator and / or been a member of advisory boards for Allergopharma, ALK-Abello, Bencard, GSK, Hermal, Novartis, Stallergenes, Stiefel. M. Deleuran is speaker, investigator and/or advisor for Leo Pharma, Pierre Fabre, MEDA and Astellas. J. Ring conducted clinical trials or research for ALK-Abello, Allergopharma, Allmirall-Hermal, Astellas, Bencard, Biogen-Idec, Galderma, GSK, Leo, MSD, Novartis, Phadia, PLS Design, Stallergenes. A. Wollenberg has received research funding and lecture honoraria from, conducted clinical trials for, or is a paid consultant to Astellas, Basilea, Galderma, GSK, Loreal, MEDA, Merck, Novartis, Pierre Fabre, MSD. Other authors declared that they have no competing interests.

## Authors’ contributions

UD coordinated and participated in the consensus process and drafted the manuscript. AW, DS, AT, TW, AO, CG, AS, MD, AMC, FG, JL, SS and JR participated in the consensus process and drafted the manuscript. All authors read and approved the final manuscript.

## Authors’ information

European Task Force on Atopic Dermatitis / EADV Eczema Task Force: O. Baadsgaard, Hellerup, Denmark; T. Bieber, Bonn, Germany; E. Bonifazi, Bari, Italy; C.A.F.M. Bruijnzeel-Koomen, Utrecht, The Netherlands; A. M. Calza, Geneva, Switzerland; M. Deleuran, Aarhus,Denmark; U. Darsow, Munich, Germany; J. De la Cuadra, Valencia, Spain; L. De Raeve, Brussels, Belgium; T.L. Diepgen, Heidelberg, Germany; P. Dupuy, Castanet Tolosan, France; G. Fabrizi, Rome, Italy; C. Gelmetti, Milan, Italy; A. Giannetti, Modena, Italy; U. Gieler, Gießen, Germany; F. Giusti, Modena, Italy; J. Harper, London, England; E.A. Holm, Copenhagen, Denmark; M. Kägi, Zürich, Switzerland; O. Kekki, Tampere, Finland; B. Kunz, Hamburg, Germany; R. Lever, Glasgow, Scotland; J. Lübbe, Geneva, Switzerland; A.B. Olesen, Aarhus, Denmark; A. P. Oranje, Rotterdam, The Netherlands; Y. de Prost, Paris, France; G. Rajka, Oslo, Norway; T. Reunala, Tampere, Finland; J. Revuz, Créteil, France; J. Ring, Munich, Germany; S. Seidenari, Modena, Italy; D. Simon, Bern, Switzerland; M. Song, Brussels, Belgium; J.F. Stalder, Nantes, France; A. Svensson, Malmö, Sweden; A. Taïeb, Bordeaux, France; D. Tennstedt, Brussels, Belgium; K. Turjanmaa, Tampere, Finland; C. Vestergaard, Aarhus, Denmark; T. Werfel, Hannover, Germany; A. Wollenberg, Munich, Germany

## References

[B1] Ring J, Przybilla B, Ruzicka T Handbook of atopic eczema 2006 2 Heidelberg: Springer

[B2] Ring J Alomar A Bieber T Deleuran M Fink-Wagner A Gelmetti C Gieler G Lipozencic J Luger T Oranje AP Schäfer T Schwennesen T Seidenari S Simon D Ständer S Stingl G Szalai S Szepietowski JC Taïeb A Werfel T Wollenberg A Darsow U Guidelines for Treatment of Atopic Eczema (Atopic Dermatitis) Part I J Eur Acad Dermatol Venereol 2012 6 1045 1060 10.1111/j.1468-3083.2012.04635.x 22805051

[B3] Manzini BM Ferdani G Simonetti V Donini M Seidenari S Contact sensitization in children Contact Dermatitis 1998 6 12 17 10.1046/j.1525-1470.1998.1998015012.x 9496796

[B4] Mortz CG Andersen KE Allergic contact dermatitis in children and adolescents Contact Dermatitis 1999 6 121 130 10.1111/j.1600-0536.1999.tb06102.x 10475509

[B5] Devillers AC Oranje AP Efficacy and safety of 'wet-wrap' dressings as an intervention treatment in children with severe and/or refractory atopic dermatitis: a critical review of the literature Br J Dermatol 2006 6 579 585 10.1111/j.1365-2133.2006.07157.x 16536797

[B6] Schnopp C Holtmann C Stock S Remling R Fölster-Holst R Ring J Abeck D Topical steroids under wet-wrap dressings in atopic dermatitis-a vehicle-controlled trial Dermatology 2002 6 56 59 10.1159/000051811 11834851

[B7] Wollenberg A Bieber T Proactive therapy of atopic dermatitis -an emerging concept Allergy 2009 6 276 278 10.1111/j.1398-9995.2008.01803.x 19076538

[B8] Van der Meer JB Glazenburg EJ Mulder PG Eggink HF Coenraads PJ The management of moderate to severe atopic dermatitis in adults with topical fluticasone propionate. The Netherlands Adult Atopic Dermatitis Study Group Br J Dermatol 1999 6 1114 1121 10354080 10.1046/j.1365-2133.1999.02893.x

[B9] Feiwel M Munro D James V Effect of topically applied 0.1% betamethasone valerate ointment on the adrenal function of children Thirteenth congress of International Dermatology 1968 Berlin: Springer 202 204

[B10] Walsh P Aeling J Huff L Weston W Hypothalamus-pituitary-adrenal axis suppression by superpotent topical steroids J Am Acad Dermatol 1993 6 501 503 10.1016/S0190-9622(08)82011-7 8349876

[B11] Bornhövd E Burgdorf WHC Wollenberg A Macrolactam immunomodulators for topical treatment of inflammatory skin diseases J Am Acad Dermatol 2001 6 736 743 10.1067/mjd.2001.117525 11606925

[B12] Ruzicka T Bieber T Schöpf E Rubins A Dobozy A Bos J A short-term trial of tacrolimus ointment for atopic dermatitis N Engl J Med 1997 6 816 821 10.1056/NEJM199709183371203 9295241

[B13] Van Leent EJ Graber M Thurston M Wagenaar A Spuls PI Bos JD Effectiveness of the ascomycin macrolactam SDZ ASM 981 in the topical treatment of atopic dermatitis Arch Dermatol 1998 6 805 809 10.1001/archderm.134.7.805 9681343

[B14] Reitamo S Wollenberg A Schöpf E Perrot JL Marks R Ruzicka T Safety and efficacy of 1 year of tacrolimus ointment monotherapy in adults with atopic dermatitis Arch Dermatol 2000 6 999 1006 10.1001/archderm.136.8.999 10926735

[B15] Meurer M Fölster-Holst R Wozel G Weidinger G Jünger M Bräutigam M Pimecrolimus cream in the long-term management of atopic dermatitis in adults: a six-month study Dermatology 2002 6 271 277 10.1159/000065863 12399676

[B16] Wollenberg A Reitamo S Girolomoni G Lahfa M Ruzicka T Healy E Giannetti A Bieber T Vyas J Deleuran M Proactive treatment of atopic dermatitis in adults with 0.1% tacrolimus ointment Allergy 2008 6 742 750 10.1111/j.1398-9995.2008.01683.x 18445188

[B17] Thaci D Reitamo S Gonzalez Ensenat MA Moss C Boccaletti V Cainelli T Van Der Valk P Buckova H Sebastian M Schuttelaar M Ruzicka T Proactive disease management with 0.03% tacrolimus ointment for children with atopic dermatitis: results of a randomized, multicentre, comparative study Br J Dermatol 2008 6 1348 1356 10.1111/j.1365-2133.2008.08813.x 18782319

[B18] Reitamo S Rustin M Ruzicka T Cambazard F Kalimo K Friedmann P Efficacy and safety of tacrolimus ointment compared with hydrocortisone butyrate ointment in adult patients with atopic dermatitis J Allergy Clin Immunol 2002 6 547 555 10.1067/mai.2002.121832 11898005

[B19] Luger T Van Leent E Graeber M Hedgecock S Thurston M Kandra A SDZ ASM 981: an emerging safe and effective treatment for atopic dermatitis Br J Dermatol 2001 6 788 794 10.1046/j.1365-2133.2001.04134.x 11298538

[B20] Diepgen TL Long-term treatment with cetirizine of infants with atopic dermatitis: a multi-country, double-blind, randomized, placebo-controlled trial (the ETAC trial) over 18 months Pediatr Allergy Immunol 2002 6 278 286 10.1034/j.1399-3038.2002.01047.x 12390444

[B21] De Benedetto A Agnihothri R McGirt LY Bankova LG Beck LA Atopic dermatitis: a disease caused by innate immune defects J Invest Dermatol 2009 6 14–30 22 19078985 10.1038/jid.2008.259

[B22] Lübbe J Secondary infections in patients with atopic dermatitis Am J Clin Dermatol 2003 6 641 654 10.2165/00128071-200304090-00006 12926982

[B23] Shah M Mohanraj M High levels of fusidic acid resistant Staphylococcus aureus in dermatology patients Br J Dermatol 2003 6 1018 1020 10.1046/j.1365-2133.2003.05291.x 12786835

[B24] Niebuhr M Mai U Kapp A Werfel T Antibiotic treatment of cutaneous infections with Staphylococcus aureus in patients with atopic dermatitis: current antimicrobial resistances and susceptibilities Exp Dermatol 2008 6 953 957 10.1111/j.1600-0625.2008.00734.x 18557929

[B25] Bunikowski R Mielke M Skarabis H Worm M Anagnostopoulos I Kolde G Evidence for a disease-promoting effect of staphylococcus aureus-derived exotoxins in atopic dermatitis J Allergy Clin Immunol 2000 6 814 819 10.1067/mai.2000.105528 10756234

[B26] Zollner T Wichelhaus T Hartung A Von Mallinckrodt C Wagner T Brade V Colonization with superantigen-producing staphylococcus aureus is associated with increased severity of atopic dermatitis Clin Exp Allergy 2000 6 994 1000 10.1046/j.1365-2222.2000.00848.x 10848922

[B27] Hauser C Wuethrich B Matter L Staphylococcus aureus skin colonization in atopic dermatitis Dermatologica Helvetica 1985 6 35 3972149

[B28] Breuer K Haussler S Kapp A Werfel T Staphylococcus aureus: colonizing features and influence of an antibacterial treatment in adults with atopic dermatitis Br J Dermatol 2002 6 55 61 10.1046/j.1365-2133.2002.04872.x 12100185

[B29] Lintu P Savolainen J Kortekangas-Savolainen O Kalimo K Systemic ketoconazole is an effective treatment of atopic dermatitis with IgE-mediated hypersensitivity to yeasts Allergy 2001 6 512 517 10.1034/j.1398-9995.2001.056006512.x 11421895

[B30] Mayser P Kupfer J Nemetz D Schäfer U Nilles M Hort W Gieler U Treatment of head and neck dermatitis with ciclopiroxolamine cream - results of a double-blind, placebo-controlled study Skin Pharmacol Physiol 2006 6 153 158 10.1159/000092596 16612143

[B31] Williams HC Grindlay DJ What's new in atopic eczema? An analysis of the clinical significance of systematic reviews on atopic eczema published in 2006 and 2007 Clin Exp Dermatol 2008 6 685 688 10.1111/j.1365-2230.2008.02906.x 18691244

[B32] Akhavan A Rudikoff D Atopic dermatitis: systematic immunosuppressive therapy Semin Cutan Med Surg 2008 6 151 155 10.1016/j.sder.2008.04.004 18620137

[B33] Berth-Jones L Finlay A Zaki I Tan B Goodyear H Lewis-Jones S Ciclosporin in severe childhood atopic dermatitis: a multicentre study J Am Acad Dermatol 1996 6 1016 1021 10.1016/S0190-9622(96)90281-9 8647967

[B34] Van Joost T Heule F Korstanje M Van-den-Broek M Stenveld H Van-Vloten W Ciclosporin in atopic dermatitis: a multicentre, placebo-controlled study Br J Dermatol 1994 6 634 640 10.1111/j.1365-2133.1994.tb13111.x 8204472

[B35] Murphy LA Atherton D A retrospective evaluation of azathioprine in severe childhood atopic eczema, using thiopurine methyltransferase levels to exclude patients at high risk of myelosuppression Br J Dermatol 2002 6 308 315 10.1046/j.1365-2133.2002.04922.x 12174104

[B36] Berth-Jones J Takwale A Tan E Barclay G Agarwal S Ahmed I Azathioprine in severe adult atopic dermatitis: a double-blind, placebo-controlled, crossover trial Br J Dermatol 2002 6 324 330 10.1046/j.1365-2133.2002.04989.x 12174106

[B37] Grundmann-Kollmann M Podda M Ochsendorf F Boehncke W-H Kaufmann R Zollner T Mycophenolate mofetil is effective in the treatment of atopic dermatitis Arch Dermatol 2001 6 870 873 11453805

[B38] Heller M Shin HT Orlow SJ Schaffer JV Mycophenolate mofetil for severe childhood atopic dermatitis: experience in 14 patients Br J Dermatol 2007 6 127 132 10.1111/j.1365-2133.2007.07947.x 17489974

[B39] Murray ML Cohen JB Mycophenolate mofetil therapy for moderate to severe atopic dermatitis Clin Exp Dermatol 2007 6 23 27 17059445 10.1111/j.1365-2230.2006.02290.x

[B40] Goujon C Berard F Dahel K Guillot I Hennino A Nosbaum A Saad N Nicolas JF Methotrexate for the treatment of adult atopic dermatitis Eur J Dermatol 2006 6 155 158 16581567

[B41] Simon D Wittwer J Kostylina G Buettiker U Simon HU Yawalkar N Alefacept (lymphocyte function-associated molecule 3/IgG fusion protein) treatment for atopic eczema J Allergy Clin Immunol 2008 6 423 424 10.1016/j.jaci.2008.06.010 18602679

[B42] Moul DK Routhouska SB Robinson MR Korman NJ Alefacept for moderate to severe atopic dermatitis: a pilot study in adults J Am Acad Dermatol 2008 6 984 989 10.1016/j.jaad.2008.02.007 18395294

[B43] Simon D Hösli S Kostylina G Yawalkar N Simon HU Anti-CD20 (rituximab) treatment improves atopic eczema J Allergy Clin Immunol 2008 6 122 128 10.1016/j.jaci.2007.11.016 18206507

[B44] Darsow U Wollenberg A Simon D Taïeb A Werfel T Oranje A Gelmetti C Svensson A Deleuran M Calza AM Giusti F Lübbe J Seidenari S Ring J for the European Task Force on Atopic Dermatitis / EADV Eczema Task Force ETFAD / EADV Eczema Task Force 2009 position paper on diagnosis and treatment of atopic dermatitis J Eur Acad Dermatol Venereol 2010 6 317 328 10.1111/j.1468-3083.2009.03415.x 19732254

[B45] Staab D Diepgen TL Fartasch M Kupfer J Lob-Corzilius T Ring J Age related, structured educational programmes for the management of atopic dermatitis in children and adolescents: multicentre, randomised controlled trial BMJ 2006 6 933 938 10.1136/bmj.332.7547.933 16627509 PMC1444870

